# MASLD and aspartame: are new studies in the horizon?

**DOI:** 10.3389/fmed.2023.1266918

**Published:** 2023-12-08

**Authors:** Consolato M. Sergi

**Affiliations:** ^1^Department of Laboratory Medicine, University of Alberta, Edmonton, AB, Canada; ^2^Children's Hospital of Eastern Ontario, University of Ottawa, Ottawa, ON, Canada

**Keywords:** fatty liver, NLRP3, cancer, aspartame, NOD-like receptor-enclosing protein 3

## Abstract

Fatty liver disease has been on the rise in the past few decades, and there is no hope that it will stop. The terminology change that has been recently proposed may not be sufficient to advocate for a reduction of steatogenic foods and a change in lifestyle. A course change may be supported by the recent labeling of aspartame sweetener as a possible carcinogenic compound by the International Association for Research on Cancer (IARC), an agency of the World Health Organization (WHO). Aspartame sweeteners and other edulcorating molecular compounds besides colorings may trigger liver cancer other than fatty liver disease, despite limited data supporting it. An essential bias in human cohort studies is indeed the exclusion of all confounding factors, which may be barely impossible for human studies. In this perspective, we suggest that the activation of the NOD-like receptor-enclosing protein 3 (NLRP3) inflammasome and the stimulation of the tumor suppression gene *TP53* may be critical in the progression from fatty liver to liver inflammation and liver cancer. Aspartame reduces a transcriptional coactivator, precisely the peroxisomal proliferator-initiated receptor-γ (gamma) coactivator 1-α (alpha) (or PGC1α). This coactivator upregulates mitochondrial bioformation, oxidative phosphorylation, respiratory capacity, and fatty acid β-oxidation. Aspartame acts in this way, probably through the activation of *TP53*. These events have been accountable for the variations in the lipid outline in serum and total lipid storage as well as for the impairment of gluconeogenesis in the liver, as supported by the downregulation of the gluconeogenic enzymes in experimental animals, and may be relevant in humans as well.

## Introduction

Undoubtedly, one of the epidemics we are facing right now is the spread of fatty liver disease in our technological society. Most countries have individuals of all ages affected with fatty liver disease (1–3). Most recently, fatty liver disease has increased in professional and lay circles because of its morbidities associated with life style changes. There has been an outcry that the most contemporary COVID-19 pandemic restrictions may have had a role in the current rate of fatty liver due to the decrease in physical activity and an increase of both high-fructose corn syrup and adulterated food and beverages during lockdowns and school closures (4). However, it is scapegoating, and probably this trend was already significant before the COVID-19 pandemic. On the other hand, there is no doubt that the decrease in physical activity and diffuse online learning may have been responsible for hijacking and incrementing this trend, making entire cohorts of youth and children overweight and even obese.

## NAFLD vs. MASLD

Liver disease is an ongoing epidemic affecting multiple countries worldwide and different ethnic groups of individuals. Most recently, a global consensus has been reached among various panels of experts, including hepatology researchers and hepatology clinicians, who changed the acronym non-alcoholic fatty liver disease (NAFLD) into metabolic dysfunction-associated steatotic hepatic disease (MASLD) from the intermediate term of original metabolic dysfunction-linked fatty liver disease (MAFLD) (5, 6). The new terminology will probably dominate in the next few years once more publications emerge. Still, we do not need to absquatulate terms for new terms when rediscovering Greek or Latin books. The new terminology recalls the Greek etymology of fat. The panelists determined fatty was considered stigmatizing and opted for a return to the Greek term “steatotic,” which arises from “steatos” or fatty (the Greek term “στέατος” is the genitive singular of στέαρ or hard fat). The terminology of steatotic liver disease will cover MASLD and MetALD (metabolic alcoholic liver disease) simultaneously. MetALD incorporates alcoholic liver disease in individuals who consume more than 210 g/week in men and more than 140 g/week in women. The term non-alcoholic steatohepatitis or NASH will be replaced by MASH, which is metabolic dysfunction associated with steatohepatitis.

MASLD individuals commonly harbor a cardiac metabolic risk factor, such as type 2 diabetes mellitus, which is not trivial and affects several organs and systems in the human organism. The comorbidities do not need to be neglected. Individuals without metabolic parameters and an unknown etiology will be classified as patients with cryptogenic steatotic liver disease (SLD). In MetALD, there is precisely a continuum in which the liver disease can be appreciated to be either ALD or MASLD leading. The new nomenclature incorporates the agreement that even moderate alcohol consumption may alter the natural progression of fatty liver disease. We hope that randomized clinical trials will soon be carried out to identify the minimal alcohol consumption that can affect metabolic dysfunction in specific individuals or ethnically different individuals. The effort involved 236 panelists. They were from 56 individual countries and took part in several rounds of online service. They used a Delphi process. Professionals and patient advocates participated in building up the new nomenclature and change well, relying on the classic “supermajority of opinion.” A few key international medical societies participated in building the new nomenclature, including the Latin American, North American, and European Associations for the Study of the Liver (ASLD, EASL, and LASL).

## Clinical aspects

**Figure 1:**
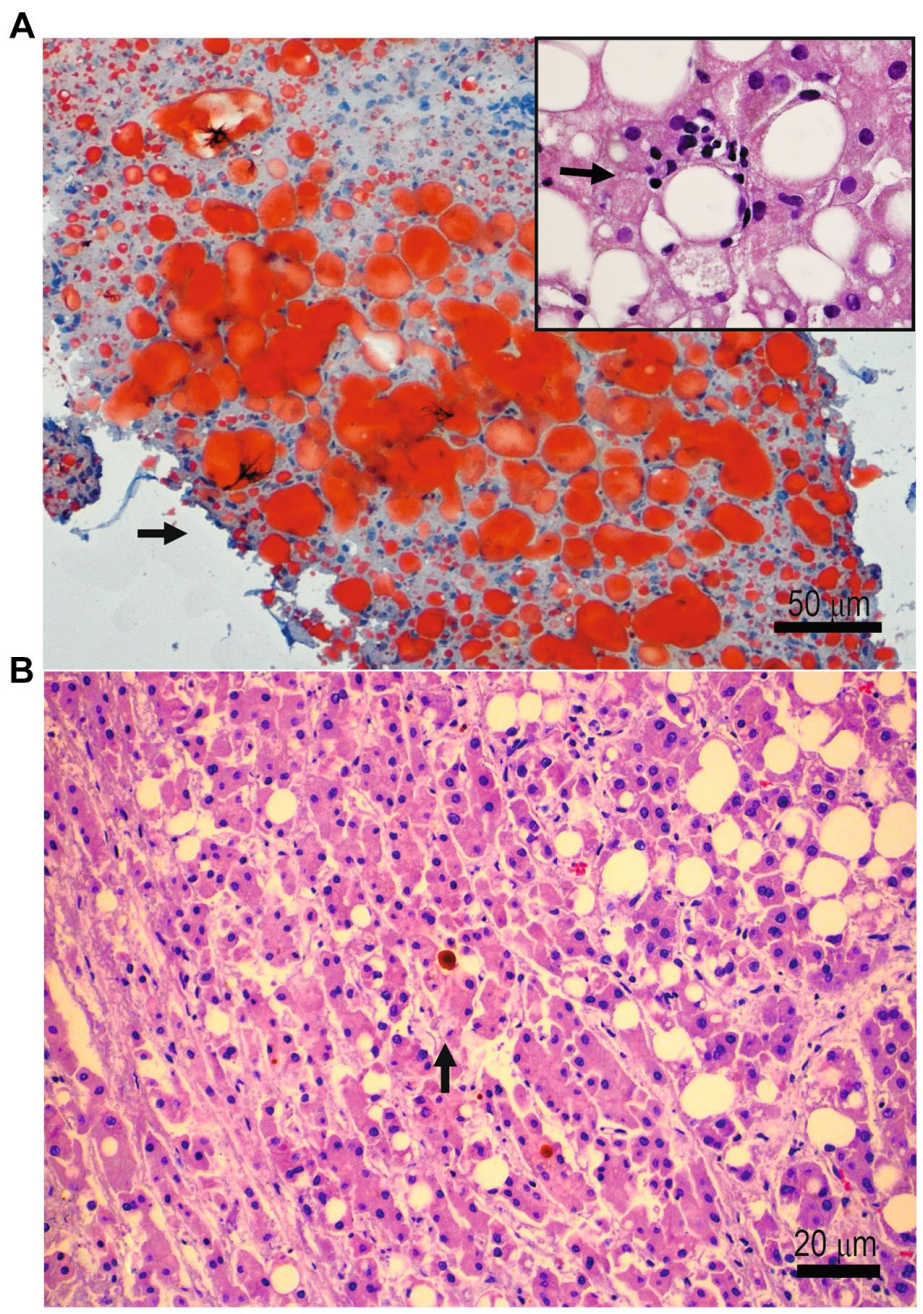
**(A)** Liver histopathology showing oil-red O-stained vacuoles in a patient affected by non-alcoholic fatty liver disease. Inset: a focus on inflammation in a patient with non-alcoholic steatohepatitis. **(B)** Liver histopathology showing neoplastic growth of cells with features of hepatocellular carcinoma in a patient with long-standing non-alcoholic fatty liver disease.

Non-alcoholic steatosis of the liver is a problematic issue because it can evolve into inflammation (NASH) and cancer (Figure 1). Steatosis is associated with metabolic syndrome in about 2/3 of cases. The increase in delivery of free fatty acids to the hepatic organ originates from inappropriate dietary fat intake, large consumption of soft drinks, increased oxidative stress in the hepatocytes, and insulin resistance. The delivery of free fatty acid to the liver and the increase of triglyceride buildup in this organ give the “fatty” change of the hepatocyte, showing histologically a multivacuolar to univacuolar transformation of the hepatocytic phenotype, which looks like a space in the hepatocyte displacing the nucleus at the periphery. NAFLD is a significant health issue, with 1/4 to 1/3 of the worldwide adult population affected (7). NAFLD can subsequently progress to NASH. There is a 20–50% risk of progression to liver fibrosis, a 30% risk of developing liver cirrhosis, and a 5% risk of developing liver neoplasm, namely, hepatocellular carcinoma (HCC) (8, 9). The presence of hyperlipidemia, hypertension, non-hypertensive cardiovascular diseases, and type 2 diabetes mellitus, in addition to NAFLD, characterizes the metabolic syndrome, which is currently harboring the name change of MASLD. It has been a matter of controversy if healthy metabolic syndrome truly exists, and several publications came out in the most recent years supporting the theory that, indeed, some individuals who are obese may also be healthy. Resveratrol is a stilbenoid (hydroxylated derivative of stilbene), i.e., a variety of phenol that occurs in nature. It is a phytoalexin. Resveratrol is produced by several plants in reaction to injury. There are multiple sources of resveratrol in food. The most salient food varieties, including resveratrol, are the skins of grapes, raspberries, mulberries, blueberries, and peanuts. Recently, a methodical search and meta-analysis was piloted to determine if cardiometabolic risk factors may be curbed by resveratrol targeting individuals with metabolic syndrome (Met-S) and individuals who are obese/healthy (O/H) (10). The first group was harboring MetS, well-defined as a gathering of obesity at the abdomen location, dyslipidemia, hyperglycemia, and hypertension in a single subject. In contrast, the second group was composed of “obese/healthy” people, i.e., healthy subjects with or without demonstrating a clinical view of obesity. Data from randomized clinical trials (RCT) studies (total: 17) comprising 651 participants were mined for analysis. Generally, resveratrol had a substantial effect on HOMA-IR, i.e., the Homeostatic Model Assessment-Insulin Resistance (HOMA-IR). It resulted in a mean difference of −0.520665 (*p* = 0.001). In Met-S, RES pointedly reduced low-density lipoprotein cholesterol (LDL-C), and total cholesterol (T-Chol) other than glucose, as discovered by the mean difference of −0.924 (*p* = 0.040), −1.246 (*p* = 0.022), and − 1.069 (*p* = 0.043), respectively. Despite some perceived heterogeneity in world people, the supplementation of resveratrol improved cardiometabolic health and clearly decreased some risk factors (HOMA-IR, LDL-C, and T-Chol) allied with cardiovascular disease (10).

## Sweeteners and fatty liver disease

**Figure 2:**
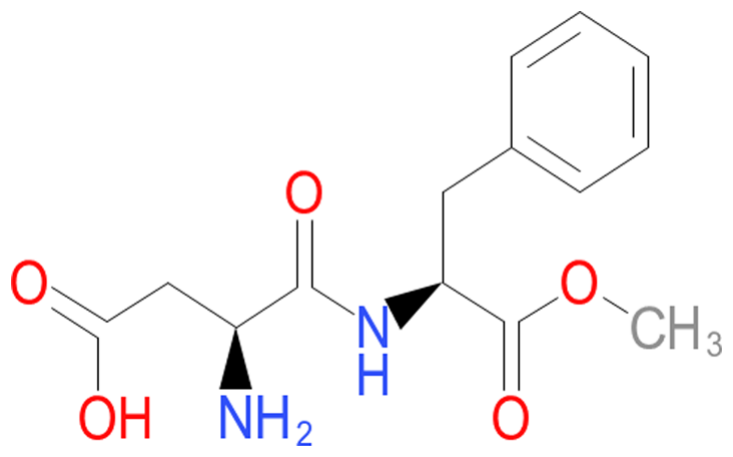
Chemical formula of aspartame.

A dramatic change occurred in the past few decades in dietary habits worldwide with the food industry’s introduction of sweeteners such as sucrose and fructose. Regular soft and fruit drinks are significant sources of high-fructose corn syrup or sugar. They increased from 3.9% of the total energy consumption in 1977 to 9.2% in 2001 (11). Thus, there is a 135% increment in just two decades. In addition to this change in dietary habits, soft drinks have increased their presence in the market. They are contained in many diet styles and have raised some health concerns. Diet soft drinks encompass aspartame sweetener (Figure 2) and often contain caramel coloring. These compounds stimulate advanced glycation end products. Aspartame sweeteners and caramel coloring are diffused in soft drinks, potentially increasing insulin resistance and inflammation (12). The increase in soft drink intake has been linked to NAFLD, a non-dependent metabolic syndrome. NAFLD patients have been seen consuming five times the quantity of carbohydrates from soft drinks compared to healthy individuals. The increase of more than one “soft” drink *per diem* has been associated with a high rate of metabolic syndrome. Some other individuals consume less than one “soft” drink daily (13). In June 2023, an assembly of 25 scientists and researchers from twelve countries met in Lyon, France, at the International Agency for Research on Cancer (IARC). They target evaluating the carcinogenesis of aspartame, among other chemicals (14). The aspartame sweetener was categorized as a compound possibly carcinogenic to humans. This classification was based on some, but limited, evidence for neoplasms in humans. The panel of 25 scientists also indicated that there was some, but limited, evidence for carcinogenicity in some experimental animals. Finally, there was little evidence of “mechanistic” data for carcinogenicity. To the best of our knowledge, the NutriNet-Sante study is leading. It is the only more extensive cohort human study that prospectively evaluated the exposure to aspartame from totally dietary sources. This cardinal investigation reported that the link with aspartame increases the mammary gland cancer risk (obesity-related) and the cumulative cancer risk. Still, they did not examine in detail the link between aspartame and liver cancer risk. On the other side, the working group recognized four prospective human cohorts that evaluated the link between artificially sweetened “soft” drinks and liver cancer risk. In three investigations, a positive association was determined between artificially sweetened drink intake and mortality due to cancer. Old studies were perused in detail and controlled for potential confounding variables. Nevertheless, the authors could not entirely rule out some confounders, and the evidence of HCC following aspartame sweetener “soft” drinks was considered to be limited.

In experimental animals, there was a split view in interpreting data for limited or sufficient evidence for carcinogenicity. Indeed, there were concerns regarding the diagnosis of lymphomas and related combinations and the likely impossible exclusion of the inflammation. However, the data from the study on animals indicated that the molecule of aspartame (methyl L-α-aspartyl-L-phenylalaninate) had clear-cut carcinogenic activity. The majority of the scientific working group pondered that the signal for oncogenesis should be considered limited concerning the appropriateness of the design and adequate reporting of the litter effect, which may have led to some false positive results. A minority of the scientific working group indicated that such concerns were indeed considered but assessed as not consistently critical. This subgroup suggested that the evidence for cancer and experimental animals was “sufficient.” An examination of the key characteristics of carcinogenicity identified that the mechanistic signal for aspartame being carcinogenic was present but still limited, despite some prominent and outstanding positive findings about genotoxicity in several investigations. These studies indicated that alterations in insulin sensitivity may have played a serious time factor. Overall, insulin sensitivity can be an important carcinogenicity factor (14).

In a most recent experimental animal study from Spain, Finamor et al. found that prolonged (long-term) aspartame administration leads ineluctably to hepatic fibrosis (15). It can elicit inflammasome activation and, subsequently, an impairment of gluconeogenesis in the liver, at least in experimental animals. They studied male Swiss mice kept in temperature- and humidity-controlled quarters. The animals were fed standard rodent chow and tap water. Aspartame produced hepatic tissue damage and a remarkable increase in transaminase levels, prompting liver fibrosis and upregulating pro-fibrotic markers, including transforming growth factor β1, collagen type 1A1, and α smooth muscle actin (SMA). Interestingly, aspartame clearly reduced the activation of nuclear factor erythroid 2-related factor 2 (Nrf2). This compound also decreased the enzymatic activity of antioxidants and caused an increase in lipid peroxidation. These events elicited the activation of the NOD-like receptor-containing protein 3 (NLRP3) inflammasome and the induction of the tumor suppression gene *TP53*. They also found that aspartame reduced the peroxisomal proliferator-activated receptor γ coactivator 1A, probably through the activation of P53. These events have been accountable for the modifications in the profile of lipids in serum and total lipid accumulation, as well as a deficiency of gluconeogenesis in the liver, as supported by the downregulation of gluconeogenic enzymes.

## NLRP3 inflammasome and cancer

NLRP3 inflammasome initiation is linked with the onset of liver cancer, particularly HCC (16). New clinical trials should target NLRP3 inflammasome activation and compounds that may be able to act on it (17). The initiation of the NLRP3 inflammasome requires two steps. First, the priming signal stimulates the nuclear factor kappa-B (NF-κB) pathway. It permits the transcription of NLRP3 and other genes. All these gene products encode some pro-inflammatory molecules (e.g., cytokines or interleukins pro-IL-1β and pro-IL-18). This initial step is generated by binding molecules of microorganisms or endogenously generated (pathogen/damage or endogenous molecular patterns, also abbreviated as PAMP and DAMP) to their receptors, labeled PRRs or “pattern recognition receptors“. The proper stimulation or second signal prompts the suitable gathering of the inflammasome (18). NLRP3 animal models may be critical to study the pathogenesis of diseases, and an animal model based on NLRP3 has been considered to be critical in studying COVID-19 properly (19). We are familiar with a rodent model because of our previous studies on inflammatory bowel disease (IBD), an idiopathic gastrointestinal disease with an inflammatory character characterized by chronicity (20, 21). *Citrobacter rodentium*, a non-invasive Gram-negative microorganism, is a natural mouse bacterium in these animal models. This microbe is generally used to explore enteric infections as well as microorganism-promoted inflammation, resembling enterohemorrhagic *Escherichia coli* infection and IBD in humans (20, 21). Mice lacking the *Nlrp3* gene (*Nlrp3*^−/−^) are critical for these studies. They are more susceptible to induced experimental IBD. *Nlrp3*^−/−^ macrophages did not respond to pathogen-associated microorganismal patterns. Formerly, we established that compensation of IL-1b in rodents (mice) missing the NLRP3 inflammasome might stimulate the clearance of *C. rodentium* by encouraging the recruitment of inflammatory macrophages early during the infection. Conversely, IL-1b overcompensation may be detrimental in wild-type animals (20). There is considerable data regarding the protagonist role of the NRLP3 inflammasome in HCC. Wei et al. (22) demonstrated that E2 supports NLRP3 inflammasome-caspase-1-dependent pyroptosis. Such an event is triggered by the inhibition of autophagy following the suppression of the AMPK/mTOR pathway in HCC cells (22). Zhang et al. (23) showed that AIF prevents the growth and metastasis of HCC cells by prompting NLRP3 inflammasome-mediated pyroptosis via the inhibition of autophagy in HCC cell lines (HepG2, Huh7, Bel7402, and SMMC 7721). Wei et al. (24) showed that E2 impedes HCC growth through the promotion of the NLRP3 inflammasome via activating the ERβ/MAPK/ERK pathway in human primary HCC samples and human HCC cell lines, including HepG2 cell lines, among others. Fan et al. (25) demonstrated that luteoloside defeats the proliferation and metastasis of HCC cells by inhibiting the NLRP3 inflammasome via decreasing the intracellular ROS (reactive oxygen species) in human neoplastic cell lines of the liver (Hep3B and SNU-449) and in an HCC animal model. Wan et al. (26) showed that miR-223-3p quashes the NLRP3 inflammasome. In this study, there is an induction of apoptosis. Finally, there is the inhibition of the proliferation of HCC cells in the Hep 3B2.1–7 cell line (26). ANI (Anisodamine or ANI) is an alkaloid extracted from Anisodus) has been suggested to promote apoptosis by suppressing the NLRP3 inflammasome in the HepG2 hepatoma cell line and HCC rodent model (27). Finally, the NLRP3 shortage in HCC augments the cytotoxicity of NK cells to HCC via the interaction of NKG2D-MIC-A. It eventually promotes the immunosurveillance of NK cells in the NK cell line NK-92, in human HCC cells, and a HCC rodent animal model (28). SHD stimulates the NLRP3 inflammasome through the promotion of the release of ROS and the suppression of the NF-κB pathway. It inhibits the growth and migration of neoplastic liver cells *in vitro* and *in vivo* (Hep3B and HepG2, among others) and in an HCC model of mice (29).

## NLRP3 inflammasome and natural compounds

Different therapeutic strategies targeting events upstream of NLRP3 inflammasome cascade activation or downstream have been evaluated for managing patients with COVID-19 (30). These treatments may be repositioned in the context of managing MASLD. Chemarin et al. enumerate colchicine, emricasan, and dapansutrile as inhibitors of NLRP3 inflammasome activation and canakinumab, anakinra, disulfiram, and dimethyl fumarate as inhibitors of the NLRP inflammasome-promoted inflammatory cascade. However, numerous natural products and chemicals can potentially target NLRP3 inflammasome cascade activation and subsequent inflammatory compounds. Some other NLRP3 inflammasome inhibitors using natural compounds are conceivably more important to highlight here. They include isoandrographolide, which targets NOD-like receptors (NLRs) and has cell differentiation-inducing and hepatoprotective properties. This molecule inhibits activation of the NLRP3 inflammasome and suppresses pneumoconiosis (silicosis) in mice. Marveloside A is one of the major biologically active components of mulberry (*Morus alba L.*). It targets TNF-α receptors and tyrosinase. Marveloside A decreases the expression of IL-1β, IL-6, and TNF-α. It has been identified that this molecule inhibits the activation of NLRP3, caspase-1, and NF-κB and the phosphorylation of ERK, JNK, and p38 and shows anti-(necro) inflammatory and anti-apoptotic effects. Muscone is probably the most important active monomer in traditional Chinese medicine. Muscon inhibits NLRP3 inflammasome activation and the upgrade of NF-κB. Muscone targets IL-6 receptors, NF-κB, NOD-like receptors (NLRs), and TNF-α receptors. Muscon markedly decreases inflammatory cytokines (IL-1β, IL-6, and TNF-α). It ultimately improves cardiac function and survival. Licochalcone B targets amyloid-β, apoptosis, and NOD-like receptors (NLRs). Licochalcone B is a compound extracted from the root of the plant *Lycopersicon esculentum*. Licochalcone B prevents amyloid-β self-aggregation, disassembles preformed Aβ42 fibers, and diminishes metal-induced Aβ42 aggregation by chelating metal ions. In addition, Licochalcone B inhibits phosphorylation of NF-κB p65 in the LPS pathway. Licochalcone B inhibits the proliferation of pulmonary carcinoma cells and induces their apoptosis. Licochalcone B inhibits the NLRP3 inflammasome by blocking the NEK7-NLRP3 interaction. Ruscogenin is an important steroidal sapogenin from *Ophiopogon japonicus* that targets NOD-like receptors. By inhibiting TXNIP/NLRP3 inflammasome activation and the MAPK pathway, Ruscogenin suppresses blood–brain barrier dysfunction caused by cerebral ischemia and exhibits marked anti-inflammatory and anti-thrombotic activity. Arglabin targets NOD-like receptors (NLRs), farnesyltransferases, and autophagy. Arglabin is also a natural product. This molecule arises from *Artemisia glabella* and is an NLRP3 inflammasome inhibitor. Arglabin exhibits anti-inflammatory and antitumor activity. The anti-neoplastic activity of algravine ensues via inhibition of farnesyltransferase, leading to activation of the RAS protogene. 4′-Methoxy resveratrol is a polyphenol. It derives from the *Dipterocarpaceae* family and has remarkable antifungal, antiandrogenic, and anti-inflammatory properties (10). 4′-Methoxy resveratrol alleviates AGE-promoted inflammation by inhibiting the RAGE-mediated MAPK/NF-κB signaling pathway and NLRP3 inflammasome activation. Soya saponin II is a saponin with substantial antiviral activity. Soya saponin II inhibits the replication of several viruses. They include herpes simplex virus 1, cytomegalovirus, influenza virus, and human immunodeficiency virus 1. Soya saponin II inhibits YB-1 phosphorylation and NLRP3 inflammasome priming. It may protect rodents from LPS/GalN-induced acute or fulminant liver failure. Picroside II is an iridoid molecular compound extracted from *Picrorhiza*. It exhibits anti-inflammatory and anti-apoptotic cellular activity. Picroside II assuages the inflammatory response in sepsis. It improves immune function by inhibiting the NLRP3 inflammasome and NF-κB pathway activation. Picroside II is an antioxidant molecular compound. It shows marked neuroprotective effects. Such effects are determined by lowering the assembly of ROS. It may be crucial in the emergency department and neurology because it protects the blood–brain barrier after a cerebral ischemia–reperfusion injury. Picroside II has pharmacological activities such as antioxidant, anti-inflammatory, and antiviral effects, in addition to immunomodulatory activities. It targets NF-κB, ROS, apoptosis, and the influenza virus.

## Conclusion

Research studies on aspartame consequences have one major factor that may affect the true impact of the investigations, i.e., dose and duration. It is extremely important to emphasize that studies in a vacuum, i.e., excluding other potential confounders, are difficult to realize, at least in humans. However, the evidence from animal studies may be comparable to that identified for other chemical compounds examined at the IARC in previous monographs.

Extensive consumption of soft drinks is prominent in determining an increase in obesity and cardio-metabolic risk factors in children, adolescents, and adults. Artificially sweetened soft drinks have been modeled as a healthier alternative to other carbonated and non-carbonated soft drinks. The evidence is that there is no protection against non-alcoholic fatty liver disease (NAFLD) using edulcorated soft drinks. We are extremely concerned about the use of sweeteners, including aspartame. It seems that aspartame, saccharine, acesulfame-K, sucralose, and neotame do not determine birth defects, but the evidence of behavioral or neurological effects in children, such as attention deficit and hyperactivity disorders, seems to be controversial. We truly hope that further research with randomized clinical trials may be set up to establish or finally exclude a potential causal relationship. In the meantime, we may suggest that diet drinks, sugar-free chewing gums, gelatins, toothpaste, and medications such as cough drops should not be part of the diet of any child, and parents should pay attention to the composition and ingredients of food given to children. In the time being, dose and duration may affect the onset of pediatric diseases. Thus, in terms of safety, aspartame should probably be banned completely, at least in pediatrics.

In addition to fatty changes in the liver and inflammation, there is a substantial risk of developing cancer. Since well-designed studies that address specific, practical, psychological, and public health issues are substantially lacking, further research is incredibly necessary. On the other hand, there is enough current theoretical conjecture to support the use of molecular compounds targeting the NLRP3 inflammasome for MASLD individuals, and randomized clinical trials are urgently warranted.

## Data availability statement

The original contributions presented in the study are included in the article/supplementary material, further inquiries can be directed to the corresponding author.

## Author contributions

CS: Conceptualization, Data curation, Formal analysis, Funding acquisition, Investigation, Methodology, Project administration, Resources, Validation, Visualization, Writing – original draft, Writing – review & editing.
